# High Sensitivity Method to Estimate Distribution of Hyaluronan Molecular Sizes in Small Biological Samples Using Gas-Phase Electrophoretic Mobility Molecular Analysis

**DOI:** 10.1155/2015/938013

**Published:** 2015-09-10

**Authors:** Lan Do, Christen P. Dahl, Susanne Kerje, Peter Hansell, Stellan Mörner, Ulla Lindqvist, Anna Engström-Laurent, Göran Larsson, Urban Hellman

**Affiliations:** ^1^Cardiology, Heart Centre and Department of Public Health and Clinical Medicine, Division of Medicine, Umeå University, 901 85 Umeå, Sweden; ^2^Department of Medical Biochemistry and Biophysics-Unit of Research Education and Development Östersund, Umeå University, 831 31 Umeå, Sweden; ^3^Department of Cardiology, Oslo University Hospital, Faculty of Medicine and K.G. Jebsen Cardiac Research Center and Center for Heart Failure Research, University of Oslo, 0327 Oslo, Norway; ^4^Department of Medical Biochemistry and Microbiology, Science for Life Laboratory, Uppsala University, 751 23 Uppsala, Sweden; ^5^Division of Integrative Physiology, Department of Medical Cell Biology, Uppsala University, 751 23 Uppsala, Sweden; ^6^Department of Medical Sciences, Uppsala University, 751 85 Uppsala, Sweden; ^7^Division of Medicine, Department of Public Health and Clinical Medicine, Umeå University, 901 85 Umeå, Sweden

## Abstract

Hyaluronan is a negatively charged polydisperse polysaccharide where both its size and tissue concentration play an important role in many physiological and pathological processes. The various functions of hyaluronan depend on its molecular size. Up to now, it has been difficult to study the role of hyaluronan in diseases with pathological changes in the extracellular matrix where availability is low or tissue samples are small. Difficulty to obtain large enough biopsies from human diseased tissue or tissue from animal models has also restricted the study of hyaluronan. In this paper, we demonstrate that gas-phase electrophoretic molecular mobility analyzer (GEMMA) can be used to estimate the distribution of hyaluronan molecular sizes in biological samples with a limited amount of hyaluronan. The low detection level of the GEMMA method allows for estimation of hyaluronan molecular sizes from different parts of small organs. Hence, the GEMMA method opens opportunity to attain a profile over the distribution of hyaluronan molecular sizes and estimate changes caused by disease or experimental conditions that has not been possible to obtain before.

## 1. Introduction

Hyaluronan (HA) is a negatively charged polysaccharide consisting of an unbranched repetitive dimer of* N*-acetyl-D-glucosamine and D-glucuronic acid [1]. It is unsulfated, in contrast to other glycosaminoglycans, and only rarely exist covalently bound to proteins. It is a polydisperse molecule, usually with an average molecular weight between 200 and 2000 kDa but it can occur up to 10^7^ Da as a single molecule [2].

In vertebrates, so far, three HA synthases are described, HA synthases 1, 2, and 3 (HAS 1–3) [3, 4] and approximately one-third of total amount of HA is turned over every day. Degradation takes place enzymatically by hyaluronidases (HYAL) or by oxidation. In humans, there are 6 genes for HYAL where 4 can give rise to functional enzymes [5].

HA is present in various forms in the tissues of the body: as a freely circulating molecule bound to hyaluronan-binding proteins, loosely associated with tissue or anchored to the cell membrane via receptors. A complex of HA bound to proteins has different properties and functions depending on its molecular weight, concentration, and the protein [6–8]. HA can also bind to cell membrane receptors, for example, CD44, which through intracellular signaling regulates cell proliferation, migration, and inflammation [9].

The various functions of HA depend on its molecular weight [10–16]. High molecular weight (HMW) HA, so-called native HA, has in some cases been shown to have opposite effect compared to fragmented HA [17, 18]. If the balance between synthesis and degradation of HA is disrupted, an accumulation of fragmented low molecular weight (LMW) HA can be formed. This is often harmful to tissues, which then cause a pathological condition.

To understand the role of HA in pathological conditions as well as in developmental processes, it is important to analyze its molecular size distribution with a reliable method suitable also for small amounts of tissues and fluids.

Gas-phase electrophoretic molecular mobility analyzer (GEMMA) (TSI Corp., MN) was originally established for globular proteins and relatively weak protein complexes [19, 20] and we have previously shown in vitro that the method can also be used for estimation of HA molecular size determination [21]. The method utilizes a differential mobility analyzer to determine the electrophoretic mobility (EMD) of a single charged molecule in air, which is proportional to the retention time and hence the electrophoretic diameter and the molecular size, of the particle. The advantage of GEMMA is the low detection level where reliable GEMMA analyses can be achieved with HA concentrations of 50 ng/mL or less, in small sample volumes of no more than 20 *μ*L [21]. The low sample concentration also ensures that the analysis is done at concentrations far below the critical concentration at which HA domain overlap occurs [22].

Due to the physical properties of HA and the shape dependence of the GEMMA method, a higher resolution is achieved in the LMW range than in the HMW range of HA [21]. The GEMMA easily separates LMW HA up to ca 100 kDa, whereas the resolution for HMW is poorer. However, in studies of biological or pathological samples this is not usually a disadvantage. The method still separates HMW from LMW HA where the resolution is high, and it is this LMW regime of HA size that shows strong cell signaling effects.

The low detection level of the GEMMA method opens new opportunities to study HA molecular size distributions from tissues in diseases that have not been possible to study before. In this paper, we demonstrate that the GEMMA method can be used for estimation of HA molecular weight distributions in biological samples with a limited amount of HA, such as from small biopsies with only few mg of available tissue.

## 2. Material

Adult white Leghorn chicken controls from a commercial population were bred and maintained at the Animal Facilities at the National Veterinary Institute in Uppsala, Sweden. A sagittal sample was collected from the middle part of the comb. These samples were frozen in −80°C. Approval from the Ethical Committee for animal experiments in Uppsala was obtained for all experiments involving the chickens (C307/11).

A heart from a wild moose was a kind gift from a local moose hunter. The heart was kept in −20°C and myocardial septum tissue samples were taken for analysis.

Human myocardial septum tissue was obtained from Oslo, Norway, from a cardiac donor who was rejected for surgical reasons. The myocardium was kept on ice for 1–4 hours before tissue sampling with subsequent freezing in −80°C. Approval for the study was obtained from the Regional Ethical Committee (Regional Etisk Komité) in Norway.

Kidney tissue was excised from adult Sprague-Dawley rats and samples from the inner medulla (papilla) were frozen in −80°C. Approval from the Ethical Committee for animal experiments in Uppsala was obtained for all experiments involving the rats (C169/11).

Except the wild moose, all animals in this project were handled by trained personnel and reared according to the guidelines from the Swedish Board of Agriculture.

## 3. Methods

### 3.1. Isolation and Purification of HA

To isolate HA from tissue samples a slightly modified protocol from Tolg et al. [23] was used. Depending on sample, between 5 and 60 mg of wet tissue was dried using a vacuum rotary evaporator (Table 1). When the tissues were completely dried, they were homogenized by grinding.

A brief summary of the protocol follows.

To digest proteins in the homogenized tissues proteinase K (Sigma-Aldrich) was used in a deferoxamine mesylate containing buffer to prevent HA degradation. HA was extracted in chloroform by liquid-liquid extraction and precipitated by ethanol (EtOH) 99%.

To digest nucleic acids, the HA containing pellets were dissolved in a buffer containing benzonase (Sigma-Aldrich) followed by digestion of chondroitin by chondroitinase ABC (Sigma-Aldrich). To avoid digestion of HA the chondroitin digestions were allowed to carry on for exactly 10 min at 37°C [24]. Chloroform extraction and precipitation by ethanol were repeated.

The crude HA-samples were purified on an anion exchange minispin column (Thermo Scientific). To remove the salt, the eluted HA fractions were dialyzed thoroughly against 20 mM of ammonium acetate at pH 8.0. The final aliquots were ready for GEMMA analysis and were subsequently diluted according to Table 1. Since the GEMMA method is not specific for HA, the purification of chicken comb was checked for impurities by GEMMA analysis before and after hyaluronidase treatment.

### 3.2. Molecular Weight Estimation with GEMMA Analysis

All HA molecular weight analyses were performed using GEMMA. Depending on the tissue origin, sample size, and initial concentration the HA samples were diluted between 1 and 100 times prior to GEMMA analysis. The final volume for all GEMMA measurements was adjusted to ca 20 *μ*L before the start of the measurements, and each GEMMA run used between 125 and 300 nL of sample. All GEMMA measurements were performed as described in Malm et al. [21] with the only exception that the differential mobility analyzer (DMA) was adjusted to scan for electrophoretic mobility of molecules with an apparent diameter between 1.72 and 54.2 nm instead of 2.55 and 25.5 nm.

## 4. Results

To show the potential of GEMMA analysis in HA research we show examples of HA size distribution in four different tissues and species, heart tissue from human and wild moose, chicken comb, and rat kidney.

**Figure 1:**
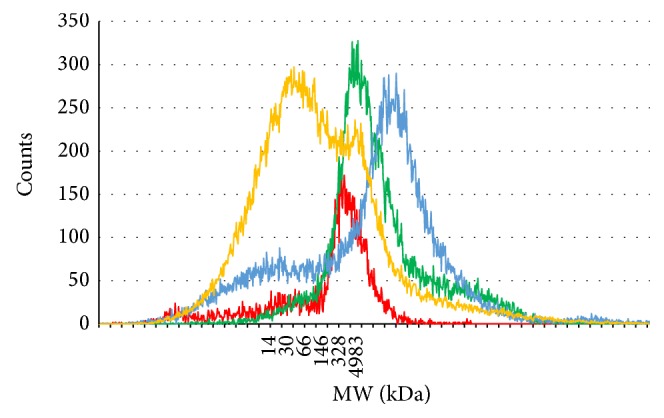
GEMMA data profile. Molecular size distribution of HA extracted from four different tissues and species, rat kidney (orange), chicken comb (green), human heart (blue), and moose heart (red), one of each. The electroforetic molecule diameter analyzed in the GEMMA was converted to molecular weight by analyzing HA standards from Hyalose L.L.C. ranging from 30 kDa to 2400 kDa samples [21]. HA from moose heart and chicken comb both showed uniform HMW HA peaks around ca 5000 kDa. HA from rat kidney showed the HMW peak and an additional broad peak with a center around 30 kDa. Human heart also showed presence of LMW HA in the same range as in rat kidney but the HMW HA content was of apparent greater size than the other three examples shown.

Weight needed for analysis was about 50 mg wet weight for heart and kidney and ca 20 mg for chicken comb samples. Purified HA extract of the heart and kidney were not necessary to dilute whereas the chicken comb HA required a 100-fold dilution prior to analysis to prevent overload of the instrument (Table 1). Differences in HA molecular size distribution were observed between all samples. The raw data from GEMMA analysis are reported as the electrophoretic mobility diameter (EMD) of the different samples. The EMD was converted to molecular weight by analyzing HA standards from Hyalose L.L.C. (Figure 1) [21]. HA from moose heart and chicken comb both showed uniform peaks around EMD 7.2 nm, which is consistent with HMW HA of ca 5000 kDa. In contrast, HA from rat kidney both showed the HMW peak seen in the moose and chicken samples, and in addition a broad peak with a center around EMD 5.2 nm, corresponding to a low molecular weight of ca 30 kDa [21]. Human heart also showed presence of LMW HA in the same range as in rat kidney but the HMW HA content was of apparent greater size than the other three examples shown.

## 5. Discussion

In this paper, some examples are given that demonstrate the ability of GEMMA to separate LMW from HMW HA in small biological samples. This opens up for the possibility to study the involvement of LMW HA in pathological conditions which have not been possible before due to lack of material.

The strength of the GEMMA compared to other methods for HA molecular size determination is the low detection level. SEC with multiangle light scattering (MALS), sedimentation techniques, flow field-flow fractionation, or gel electrophoresis usually has a detection limit of >0.01 mg/mL [25–28]. Since the GEMMA method is at least 200 times more sensitive, it is possible to analyze very small amounts of tissues, for example, comparing left and right wall in a rat heart. GEMMA can therefore be used to estimate the molecular size distribution of HA in samples with very low concentrations, something that is crucial when extracting HA from small tissue samples. However, if a more exact molecular weight determination is needed and enough material is available, then, for example, gel electrophoresis is better suited [29, 30].

As expected, moose cardiac tissue and adult chicken comb showed HMW HA content around 5000 kDA. In contrast, besides the native HMW HA seen in the inner medulla of rat kidney, a large fraction of LMW HA around 30 kDa was also found. Both overexpression of hyaluronidase 2 or hyaluronidase 1 deficiency could potentially lead to accumulation of fragmented LMW HA as seen for this sample. However, in the kidney the amount of HA changes rapidly according to body hydration, and an extensive turnover of HA in a dehydrated kidney is to be expected [31]. Thus, since the tissue analyzed within this study was from a dehydrated rat, the large fraction of fragmented HA is anticipated.

Analysis of HA standards and ladders from Hyalose L.L.C. showed that also the biggest molecular weight (6 MDa) did not have an EMD over 9 nm (data not shown). The GEMMA spectrum of the human heart sample showed a peak center at EMD ca 9 nm, indicating abnormally large HA. This could possibly be cross-linked HA molecules or HA cross-linked with proteins. The latter case would indicate a protection of the crosslinking protein from degradation during the extraction process by HA shielding it.

We have seen this very high molecular weight HA in samples from very young chicken combs (data not shown). Thus, it is likely that this very high molecular weight HA is extracted only from certain types of tissues, for example, human heart and young chicken comb tissues which excludes the extraction process as source of the crosslinking. The very high molecular weight HA in the young chicken comb is degraded by hyaluronidases but possible trace amount of some other molecules forcing crosslinking could hide in the baseline noise. This highlights the need to develop a reliable HA specific affinity extraction which would be useful for all variants of HA molecular size determination methods.

## 6. Conclusion

Up to now, it has been very difficult to study diseases with pathological changes in the extracellular matrix where availability is low or tissue samples are small in size. Difficulty to obtain large enough biopsies for HA analysis from tissues of human disease or animal disease models has in many cases restricted the study of HA in such diseases. The low detection level of the GEMMA method now allows for studies of diseases where HA is involved. In addition, the method allows for estimation of the distribution of HA molecular sizes from different parts of small organs, for example, rat kidney (Figure 1). Hence, the GEMMA gives an opportunity to attain a profile over the distribution of HA molecular weight and estimate changes caused by disease or experimental conditions that has not been possible to obtain before.

## Figures and Tables

**Table 1:** Average weight of a series of tissue biopsies used for HA isolation and subsequent GEMMA analysis. The purified chicken comb sample contained so much HA so it needed to be diluted before analysis in the GEMMA.

	Wet weight (mg)	Dry weight (mg)	Sample dilution
Heart (*n* = 24)	52	12	1x
Kidney (*n* = 15)	54	9	1x
Chicken comb (*n* = 10)	19	2,5	100x
